# Vitamin D as a Primer for Oncolytic Viral Therapy in Colon Cancer Models

**DOI:** 10.3390/ijms21197326

**Published:** 2020-10-03

**Authors:** Sang-In Kim, Shyambabu Chaurasiya, Anthony K. Park, Seonah Kang, Jianming Lu, Yanghee Woo, Hongwei Holly Yin, Zhirong Yin, Yuman Fong, Susanne G. Warner

**Affiliations:** 1 Department of Surgery, City of Hope Comprehensive Cancer Center, Duarte, CA 91010, USA; skim@coh.org (S.-I.K.); schaurasiya@coh.org (S.C.); sekang@coh.org (S.K.); jlu@coh.org (J.L.); ywoo@coh.org (Y.W.); yfong@coh.org (Y.F.); 2 Department of Hematologic Malignancies Translational Science, Beckman Research Institute, Comprehensive Cancer Center, Duarte, CA 91010, USA; apark@coh.org; 3 Molecular Pathology Core, Shared Resources, City of Hope Comprehensive Cancer Center, Duarte, CA 91010, USA; hoyin@coh.org; 4 Department of Pathology, Shared Resources, City of Hope Comprehensive Cancer Center, Duarte, CA 91010, USA; zhyin@coh.org

**Keywords:** CF33, orthopoxvirus, virotherapy, colon cancer, vitamin D

## Abstract

Oncolytic viroimmunotherapy is an exciting modality that can offer lasting anti-tumor immunity for aggressive malignancies like colon cancer. The impact of oncolytic viruses may be extended by combining them with agents to prime a tumor for viral susceptibility. This study investigates vitamin D analogue as an adjunct to oncolytic viral therapy for colon cancer. While vitamin D (VD) has historically been viewed as anti-viral, our in vitro investigations using human colon cancer cell lines showed that VD does not directly inhibit replication of recombinant chimeric poxvirus CF33. VD did restrict growth in HT29 but not HCT116 human colon cancer cells. In vivo investigations using HCT116 and HT29 xenograft models of colon cancer demonstrated that a VD analogue, calcipotriol, was additive with CF33-based viral therapy in VD-responsive HT29 but not in HCT116 tumors. Analyses of RNA-sequencing and gene expression data demonstrated a downregulation in the Jak-STAT signaling pathway with the addition of VD to viral therapy in HT29 models suggesting that the anti-inflammatory properties of VD may enhance the effects of viral therapy in some models. In conclusion, VD may prime oncolytic viral therapy in certain colon cancers.

## 1. Introduction

Colorectal cancer is the third leading cause of cancer death in the United States [1]. Even patients with early-stage disease are at risk for recurrence, with up to 50% of patients with stage III or less disease at risk of recurring [2]. For metastatic disease, 5-year survival remains low and treatments are dependent upon cytotoxic chemotherapies that are rarely curative [1,3,4]. While curative surgical treatments are available for some stage IV patients, most will ultimately succumb to their disease [5]. While some anti-tumor immune success has been seen with immunotherapy (specifically immune checkpoint inhibitors) in patients with microsatellite instability (MSI)-high colorectal cancer (CRC), responses are inconsistent [6]. Thus, novel immunogenic treatment strategies are needed to confer anti-tumor immunity alongside tumor destruction for patients with an immunosuppressed tumor microenvironment (TME). The ideal treatment for non-immunogenic colon cancers will likely involve a multi-modal approach of stromal modulation to allow delivery of targeted therapies, efficient tumor cell destruction and recruitment and activation of immune cells in the TME to establish anti-tumor immunity.

Oncolytic viruses (OVs) infect, replicate in and kill cancer cells, leaving normal cells unharmed [7]. Capable of rapidly recruiting immune and inflammatory cells, viroimmunotherapy is gaining traction as a clinically useful treatment modality, particularly in immunosuppressed tumors devoid of effector immune cells [8]. We have created a recombinant orthopoxvirus as previously described [9,10,11,12,13] and have demonstrated pre-clinical promise against colon cancer [11,12].

Our group and others have demonstrated additive/synergistic effects of combining OVs with immune checkpoint inhibition [13,14,15,16]. Specifically, our group has used a CF33-derivative, CF33-hNIS-ΔF14.5 in combination with systemically delivered anti-PD-L1 to induce durable tumor-specific cure in a breast cancer model [13]. However, in immunosuppressed tumors with substantial fibrosis, delivery to sequestered tumor cells may still represent a cure-limiting factor for immunogenic therapies like OVs [17,18]. Therefore, in these types of tumors TMEs need to be primed for optimal cytotoxic treatment or immunotherapy. Indeed, Sherman et al., showed that modulation of dense pancreas stroma enhances the delivery of chemotherapeutics [19]. Similarly, we questioned whether vitamin D (VD) could favorably prime a colon cancer microenvironment for CF33-based OV treatment.

VD analogues have long been studied as adjuncts to CRC treatment. Higher serum levels of VD have been associated with improved outcomes in colorectal cancer in some studies [20,21]. Recently reported results of the Phase II SUNSHINE trial showed that high-dose VD supplementation in stage IV colon cancer patients receiving modified FOLFOX6 plus bevacizumab chemotherapy was associated with a decreased likelihood of disease progression or death [21]. This is prompting a Phase III trial of vitamin D3 as an adjunct to chemotherapy and bevacizumab (NCT04094688). While it is difficult to understand the pleiotropic effects of VD on colon cancer, VD is capable of TME remodeling in colon cancer and several other solid tumors [22,23,24]. Thus, we hypothesized that VD would enhance the efficacy of oncolytic viral therapy in colon cancer models in vitro and in vivo.

## 2. Results

### 2.1. VD Analogue Does Not Affect Viral Replication but Reduces Cellular Proliferation in Vd-Responsive Cells

To test the effect of VD on replication of CF33-derivatives, we examined viral infection in vitro with and without the synthetic D3 derivative, calcipotriol. We tested the human colon cancer cell lines—HT29 and HCT116 with well-characterized stability to CF33 infection [11,12]. Calcipotriol was chosen due to its potency, non-hypercalcemic in vivo activity and proven stromal modulation in other cancers [19]. To test the effects of VD as a primer as well as its direct effect on viral replication, in vitro treatment groups included human colon cancer cells that had been pre-treated with VD, which then continued (concurrent treatment) or was held (pre-treatment) during viral infection.

**Figure 1:**
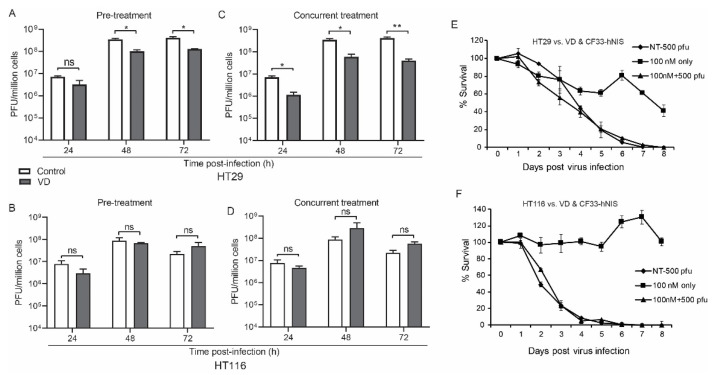
Vitamin D (VD) analogue does not directly affect viral replication. Human colon cancer cells HT29 (**A**) and HCT116 (**B**) were pre-treated with VD or phosphate buffered saline (PBS) for 12 and 14 days respectively and then infected with CF33-hNIS at a multiplicity of infection (MOI) of 0.01 and analyzed daily for 3 days via standard viral plaque assay. Abrogated viral replication in VD-responsive HT29 cells (**C**) versus non-responsive HCT116 cells (**D**) is still more pronounced when cells were concurrently treated and that treatment was continued after viral infection. Cytotoxicity assays over extended period of time with 6 days of VD pre-treatment followed by virus infection alone at a standardized dose rather than MOI are shown for HT29 (**E**) and HCT116 (**F**) cells. Stat = non-paired *t*-test, * *p* < 0.05, ** *p* < 0.01, error bars = standard deviation, ns = non-significant.

CF33-hNIS replication was unaffected at 24 h and slightly abrogated at 48 and 72 h in the HT29 but not the HCT116 VD pre-treatment group (Figure 1A,B).

Viral growth was again mildly abrogated in the concurrent treatment group for HT29 but not HCT116 cells (Figure 1C,D).

While the virus appears to induce more rapid cell death in HCT116 cells, cytotoxicity did not change in either cell line with the addition of VD to CF33-hNIS virus at a multiplicity of infection (MOI) of 0.1, likely secondary to the potency of CF33-hNIS (Figure 1E,F). However, some cell death was observed in HT29 cells treated with VD alone (Figure 1E).

### 2.2. The Differences Seen in Viral Replication in HT29 Cells with and without VD Was Attributed to the Baseline VD-Responsive Nature of HT29 Cells

**Figure 2:**
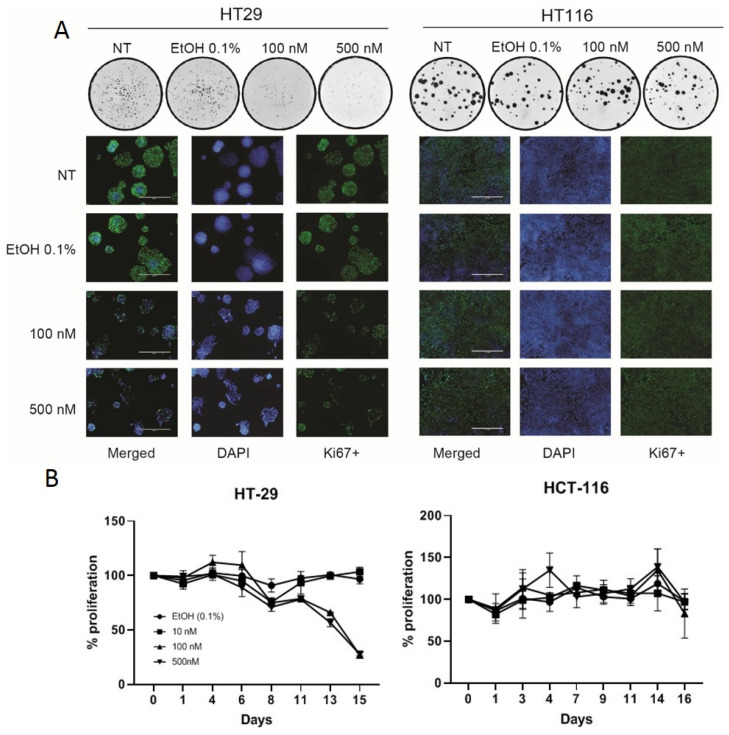
Vitamin D analogue slows cellular proliferation in VD-responsive HT-29 cells. Colony assays and ki-67 staining confirm that VD abrogates cell proliferation in HT29 but not HCT116 cells (**A**). Colony assay demonstrated in circles is representative 1x photographs of 6-well plates. White scale bars in ki-67 staining merged photos measure 400μm and reflects scale for all pictures. Cell growth curves with VD treatment versus control suggest HT29 cellular proliferation is blunted following VD dosing whereas HCT116 proliferation is unaffected (**B**).

Other authors have shown VD to cause cell-cycle arrest in HT29 cells [25,26]. We confirmed this using a colony-forming assay and ki67 staining, both of which demonstrated that VD slows the growth of HT29 cells but not HCT116 cells (Figure 2A). This was further confirmed with cell growth curves using various doses of VD versus control (Figure 2B).

Given the ability of VD to slow the growth of HT29 and the inability of VD to change replication of CF33-hNIS in the VD-unresponsive HCT116 cells, it is highly likely that the slight reduction in virus yield observed in VD-treated HT29 cells at later time points (48 and 72 h) is due to a lower number of cells for virus amplification. Taken together, these in vitro experiments suggest that VD may cause a reduction in virus yield in VD-sensitive cells through a decrease in cell growth but that VD has no direct effect on the replicative capability of CF33-derivatives.

### 2.3. VD Potentiates Oncolytic Viral Replication and Increases the Survival of Mice Bearing VD-Responsive Xenograft Tumors

To assess whether VD has any effect on OV efficacy in vivo, we used CRC xenograft tumors created from VD-responsive HT29 cells and VD non-responsive HCT116 cells.

**Figure 3:**
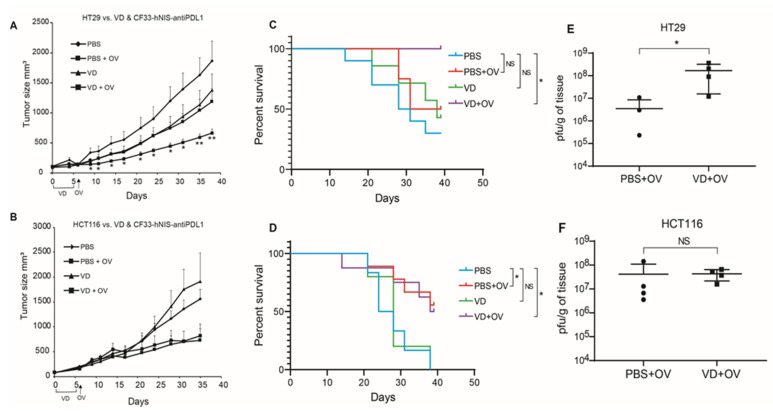
VD analogue (VD) potentiates Oncolytic virus (OV) replication and enhances survival in VD-responsive xenografts. Nude mice bearing flank tumors measuring an average of 160 mm^3^ of either VD-responsive HT29 or VD-non-responsive HCT116 were treated with IT PBS alone, IP PBS for 5 days + IT OV (CF33-hNIS-antiPDL1; 1e4 pfu), IP VD alone for 5 days (100 μg/kg), IP VD alone for 5 days + IT OV (CF33-hNIS-antiPDL1; 1e4 pfu). Tumor volumes are shown for HT29 (PBS *n* = 10; PBS + OV *n* = 8; VD *n* = 7; VD + OV *n* = 6) (**A**) and HCT116 (PBS *n* = 6; PBS + OV *n* = 9; VD *n* = 5; and VD + OV *n* = 8) (**B**) showing additive effects of VD in HT29 but not HCT116. Survival curves are shown for HT29 (**C**) and HCT116 (**D**) using humane endpoint of 1000 mm^3^ tumor volume for terminal event. 39 days following tumor treatment initiation, all mice were euthanized and tumor titers performed using standard plaque assay, showing increased viral replication in HT29 VD + OV combination group (**E**) but not HCT116 VD + OV combination group (**F**). Statistics for A & B = Welch’s *t*-test, error bars = SEM; * *p* < 0.05; Statistics for C & D = Log-rank (Mantel-Cox) test; Statistics for E & F = Mann-Whitney test * *p* < 0.05.

HT29 xenografts showed significantly blunted tumor growth when mice were pre-treated with VD before viral infection (Figure 3A). Whereas HCT116 xenografts were unaffected by the addition of VD (Figure 3B).

This translated into enhanced survival in combination-treated HT29 xenografts but not HCT116 (Figure 3C,D).

Interestingly, upon euthanasia at day 39 post-treatment, we observed higher titers of the virus in HT29 tumors treated with VD and virus compared to those treated with virus alone (Figure 3E). Viral titers were similar in the HCT116 tumors between treatment groups (Figure 3F).

### 2.4. VD Blunts Inflammation and Promotes Tumor Destruction After Viral Infection in VD-Responsive HT29 Xenografts

**Figure 4:**
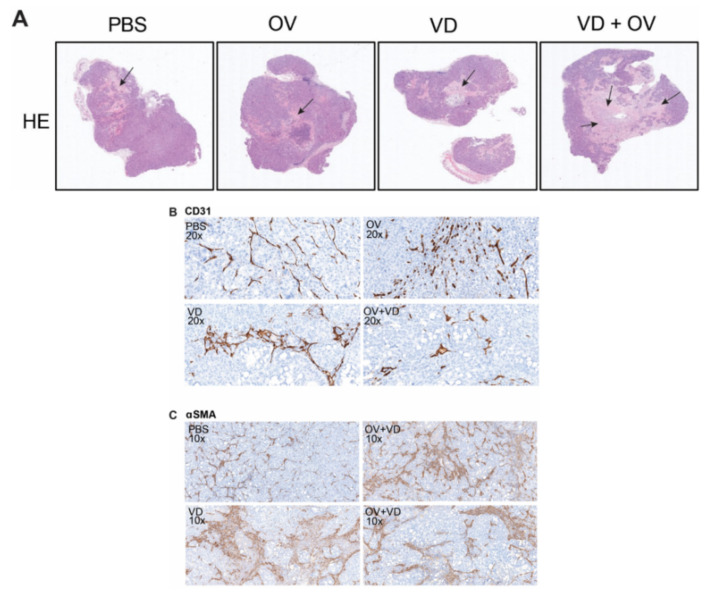
VD analogue (VD) enhances tumor necrosis and alters tumor fibrosis and vasculature. Hematoxylin and eosin (H&E) staining shows representative histological differences between groups with black arrows marking areas of tumor necrosis. Scale bars = 500μm (**A**). Sections analyzed and graded by pathologist showed differing intratumoral vascular density (**B**) corresponding with varied status of intratumoral stromal cells (**C**).

Histopathologic examination of representative tumors from each treatment group was performed by a veterinary pathologist. Representative tumor cross-sections following hematoxylin and eosin staining are shown (Figure 4A). Magnified views demonstrate altered morphology and density of CD31+ tumor vasculature in necrosis-free areas (Figure 4B) as well as α-SMA+ cancer-associated fibroblasts representing degree of tumor fibrosis differs between treatment groups (Figure 4C).

Substantial tumor necrosis and destruction was noted in the VD and virus combination treatment group. When compared to the OV alone treatment group, the stroma was less dense with evidence of fewer α-SMA + stromal cells and fewer CD31+ tumor blood vessels and less inflammation and lymphocyte infiltration in the tumors treated with a combination of OV and VD. Table 1 demonstrates a summary of pathological changes according to independent pathologist grading.

### 2.5. RNA-Sequencing Analysis of HT29 Treatment Groups Shows Downregulated Jak-STAT Pathway Signaling When Comparing Virus Alone to VD Combined with Virus

To further understand the differences between treatment groups, single-cell RNA-sequencing analysis was performed to compare samples from HT29 tumors from each treatment group.

**Figure 5:**
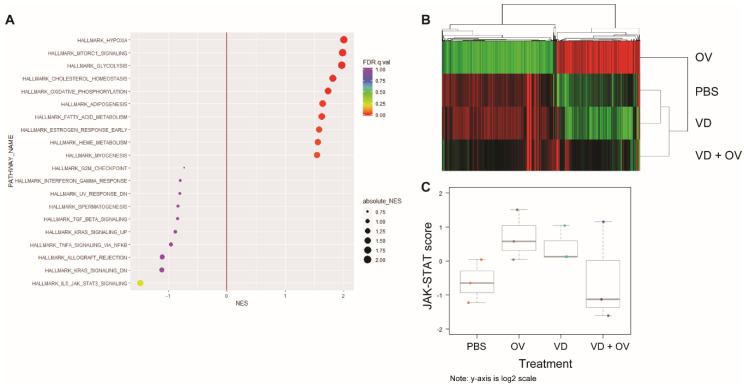
RNA sequencing shows downregulated Jak-STAT signaling in combination VD + OV group. Single-cell RNA sequencing analysis of HT29 tumor treatment groups’ expression of hallmarks of cancer gene signatures showed that when compared to OV alone, OV + VD shows downregulated Jak-STAT signaling and upregulated hypoxia, mammalian target of rapamycin (mTOR) and glycolysis signaling (**A**) Heatmap demonstrates that genes in virus alone group that have near opposite signatures to PBS and VD alone groups. VD + Virus RNA degraded due to tumor destruction but indicates a more heterogenous signature (**B**) Using NanoString nCounter analysis to validate these findings, we confirmed that Jak-STAT signaling was downregulated in the OV + VD group compared to OV alone as indicated by Jak-STAT pathway score differences (**C**). The pathway scores are used to summarize the data from a pathway’s genes into a single score, they are calculated using Principal Component (PC) analysis, they are plotted to show how they vary across different values of tested conditions. Y-axis is at log_2_ scale and increased score indicates increased overall expression in that pathway.

Figure 5A shows the most commonly upregulated genes between the virus alone and VD + virus treated groups. When comparing the Kyoto Encyclopedia of Genes and Genomes (KEGG) Hallmarks of cancer signatures between VD + virus and virus alone group, we noted downregulation in DNA-sensing, chemokine signaling and cytokine receptor interaction pathways and upregulation in gluconeogenesis, oxidative phosphorylation and amino sugar and nucleotide sugar metabolism. We also noted a significant downregulation of Jak-STAT signaling.

A heatmap representing gene expression between virus alone, PBS, VD and VD + virus treatment groups is shown, demonstrating little difference between PBS and VD alone (Figure 5B).

Due to concern for RNA quality secondary to the efficacy of combination treatment and tumor cell death in this group, we repeated this analysis using the nCounter system which does not require an amplification step. We were also able to increase the number of samples analyzed to three per group using this method. Corresponding pathway scores are presented in Figure 5C for this analysis demonstrating downregulated Jak-STAT signaling in the VD + virus combination groups compared to virus or VD alone groups.

## 3. Discussion

Herein, we show that replication of the CF33 platform-derived viruses is unimpeded by VD in vitro. In a cell line that is VD-responsive at baseline, viral replication efficiency is altered due to the speed of cellular replication but this appears to be entirely mediated by the effects of VD on the cells. This study is novel in its combination of VD and OV. Further novelty lies in the demonstration of increased viral replication in VD-responsive tumors. The initial intent of this project was to explore VD as a primer for virotherapy. What we found in these models was that in VD-responsive tumors, VD may be additive with OV therapy.

VD has been unexplored as an oncolytic viral adjunct because it has long been considered an anti-viral agent [27]. Thus, how VD affects oncolytic viral therapy for CRC is unknown. In this study, we have demonstrated that VD per se does not affect the oncolytic properties of CF33. This also further aligns with data suggesting that VD does not affect the life cycle of the hepatitis C virus (HCV) but instead enhances the IFN-α-mediated inhibition of HCV replication [28]. One other group has shown that while VD does affect adenoviral replication, this is highly dependent upon the type of virus within a given viral family [23,29]. We further showed that in HT29 xenografts, viral titer was higher in VD treated groups at euthanasia many days following viral injection. Given that VD is known to induce cell-cycle arrest in HT29 cells [25], it is possible that virus was able to usurp the machinery of alive, non-dividing HT29 cells for longer, thereby resulting in a higher viral load within these tumors. This represents and interesting avenue for future study using fresh human samples or other models more closely representative of the colon cancer tumor microenvironment.

The only other study involving VD and OV, incorporated an osteocalcin promoter to control an essential gene (E1B) in an oncolytic adenovirus followed by the addition of vitamin D to increase the promoter activity. While the osteocalcin promoter allowed the cancer-specific oncolytic activity of the virus, the promoter strength was found to be low even after the addition of VD [30]. In the present study, histopathologic analysis revealed more tumor death and necrosis in the VD + OV combination treatment group along with decreased inflammatory cell infiltration. While inflammation in a nude mouse model cannot be immediately extrapolated to humans, our findings provide the basis for further study of VD as an OV adjunct in syngeneic models and additional mechanistic studies as to what microenvironment changes might make some tumors more susceptible than others to VD and OV combination treatment. Due to the relatively non-toxic nature of VD, future findings could inform the clinical trial design to test a combination of VD and OV in colon cancers.

With its pleiotropic effects across TME, there is speculation that VD balances auto-immunity and inflammation, potentially rendering tumors more susceptible to immunotherapy, including oncolytic viral therapy [5,10]. This may be reflected in our findings that the addition of VD to virus treatment resulted in the downregulation of Jak-STAT pathways from both single-cell RNA-sequencing and nCounter analysis. These findings are aligned with those of other investigators who have shown VD decreases Jak-STAT activation in both cancer cell lines and murine models of autoimmune disease [31,32,33]. This also fits well with findings of Lange et al., demonstrating that inactivated VD receptor enhanced response of hepatocellular carcinoma to IFN-α treatment via the Jak-STAT pathway [28]. Given OV’s propensity to promote type I IFN signaling within otherwise cold microenvironments, further attention is warranted to understanding how VD might affect anti-tumor viral efficacy in different settings.

One limitation of this study is that HCT 116 xenografts have previously been shown to be highly susceptible to CF33-based infection [11,12]. Thus, it is possible that any additive effects of VD would be muted by viral efficacy. Additional limitations are the absence of immunocompetent model verification. However, while vitamin D receptor gene coding is similar between mice and humans these studies would be of questionable relevance given the difference in murine VD immune response from humans [34]. Thus, we believe immunocompetent murine models would be unlikely to recapitulate the exact efficacy of OV combined with VD in human cancer treatment. Given the relatively non-toxic and inexpensive nature of VD treatment, this study adds an intriguing possible dimension to future clinical applications of oncolytic viral therapy. Our findings of decreased fibrosis and increased vasculature along with lower inflammatory signaling in the VD treatment groups is aligned with those of other investigators examining VD-induced stromal alterations in pancreas cancer [19]. Thus, beyond the immune-inflammatory modulation potential, future studies will build on the concept of VD as a stromal modulator to examine whether it facilitates better dissemination of OVs in tumors with dense stroma.

In conclusion, this study demonstrates the additive potential of VD to enhance the effects of OV treatment in a VD-responsive colon cancer model. Further studies examining this intriguing and easily translatable concept in additional colon cancer models and in other solid tumors are ongoing.

## 4. Materials and Methods

### 4.1. CF33 Chimerization and hNIS, Anti-PD-L1 or ΔF14.5 Cloning

The chimerization, cloning, competitive selection and sequence of CF33 backbone virus have been described previously [10,11,13,35]. In brief, 9 strains of orthopoxvirus were co-infected and 100 resulting viral plaques were clonally isolated and purified. CF33 was selected for its potency across solid tumor types. Subsequent manipulations of CF33 backbone virus have ensued. Insertion of the hNIS expression cassette or firefly luciferase under the control of the vaccinia H5 synthetic early (SE) promoter at the *J2R* locus has also been described [11,12], as has the deletion of the *F14.5L* gene [13,35] and insertion of the anti-PD-L1 transgene at the *F14.5L* locus [16]. Previous experiments have demonstrated minimal-to-no differences in viral function with in vitro between parent CF33 and CF33-hNIS or CF33-hNIS-anti-PD-L1 or CF33-hNIS-ΔF14.5 [12,16]. Therefore, they are often used interchangeably. When CF33-hNIS-anti-PD-L1 was used, assays examining tumor lysates confirmed virus-specific production of anti-PD-L1 (Supplemental Figure S1).

### 4.2. Cell Lines and Mice

HCT116 (RRID: CVCL_0291), HT-29 (RRID: CVCL_0320) and African green monkey kidney fibroblasts—CV-1 (RRID: CVCL_0229) cell lines were purchased from American Type Culture Collection (ATCC, Manassas, VA, USA). All Human colorectal cell lines were cultured in McCoy’s 5A medium (Gibco, Gaithersburg, MD, USA). CV-1 cells were maintained in Dulbecco’s modified Eagle’s medium (Corning, Corning, NY, USA). All cells were supplemented with 10% fetal bovine serum (FBS) and 1% antibiotic-antimycotic solution, both purchased from Corning (Corning, NY, USA). The cells were maintained in a humidified incubator at 37 °C with 5% CO_2_. All cell lines were tested for mycoplasma before each experiment initiation and efforts were made not to perform experiments past 15 passages. Six-week-old Hsd:Athymic Nude-Foxn1nu female mice (Envigo, Indianapolis, IN, USA) and 8–12 weeks old C57Bl/6J female mice (Jackson Laboratories, Bar Harbor, ME & Charles River, Wilmington, MA, USA, RRID: IMSR_JAX:000664, RRID: IMSR_CRL:027) were used for experiments. All mice were purchased and acclimatized for seven days. Mice were maintained in a biosafety containment level 2 facility within our vivarium where the environment was temperature and light controlled with 12-h light and 12-h dark cycles and food and water were ingested ad libitum. All animal experiments were performed with the approval of the City of Hope Institutional Animal Care and Use Committee (IACUC, #15003, 18 March 2019).

### 4.3. Tumor Models

For the athymic nude mouse models, flank tumors were generated by injecting 2–3 × 10^6^ HT29 or HCT116 cells in a total of 100 µL PBS containing 50% matrigel to create each tumor. When the average tumor size approached greater than 100 mm^3^, mice were divided into experimental groups and treated with 100 μg/kg of Vitamin D analogue (VD; Calcipotirol (Tocris, Cat. No. 2700)) for 5 days by intraperitoneal (IP) injection. After 5 days VD treatment, mice were treated with 10^4^ PFU of CF33-hNIS-anti-PD-L1 in 50 µL PBS by intratumoral (IT) injection. The average tumor size was approximately 160 mm^3^. All tumors measuring greater than 200 mm^3^ (HT29) or 250 mm^3^ (HCT116) at virus treatment initiation were excluded. Iterations of this experiment were performed with similar results. For all experiments, tumor measurements and mouse weight were monitored twice weekly using calipers to calculate tumor volume, V (mm^3^) = (1/2) × A^2^ × B, where A is the shortest and B is the longest measurement.

### 4.4. Viral Growth Assays

HT29 and HCT116 cells were pre-treated with 100 nM VD for 12 days. Medium containing 100 nM of VD changed every 3 days. VD pre-treated and non-treated cells were seeded in 6-well plates (Corning, Corning, NY, USA) at 5 × 10^5^ cells/well and incubated. In the concurrent treatment group, cells were continuously treated with 100 nM VD. The following day, cells were infected at an MOI of 0.01 plaque-forming unit (PFU) per cell with CF33-hNIS in a total volume of 0.5 mL medium containing 2.5% FBS. After 1 h, the inoculum was aspirated and 2 mL of fresh medium was added to each well and plates were returned to the incubator. At the indicated times, cell lysates were collected by scraping and virus titers were determined by standard plaque assay as described previously [12,13].

### 4.5. Cytotoxicity Assays

Human colorectal cancer cell lines HT29 and HCT116 were plated at 500 cells per well in 96-well flat-bottom plates. Following experimental conditions, cells were treated with 100 nM of VD and incubated for 6 days before virus infection. The following day, cells were infected with 500 PFU of CF33-hNIS. For the next 8 days, cell survival was determined by comparing the absorption of infected cells to mock-infected cells after 2 h incubation with CellTiter 96^®^ AQueous One Solution Cell Proliferation Assay per manufacturer protocol (Promega, Madison, WI, USA).

### 4.6. Immunohistochemistry (IHC)

Tumors harvested at the time of sacrifice were fixed with 10% formalin. Subsequently, tumors were paraffin-embedded and 5 µm thick sections were obtained. The slides were deparaffinized and were then stained on Ventana Discovery Ultra (Ventana Medical Systems, Roche Diagnostics, IN, USA) IHC automated stainer. Hematoxylin and eosin (H&E), CD31, alpha-SMA staining and pathological analysis were performed by the Pathology Core at City of Hope. Images were obtained using the Ventana iScan HT (Roche, Basel, Switzerland).

### 4.7. RNA-Sequencing

HT29 tumors were harvested at 39 days post virus infection. Total RNA from the tumor was extracted using RNeasy Mini kit (Qiagen, Germantown, MD, USA). RNA sequencing and analysis were performed at Integrative Genomics Core at City of Hope. Sequencing libraries were prepared with Kapa mRNA HyperPrep kit (Roche) and sequenced on a HiSeq 2500 (Illumina, San Diego, CA, USA). The RNA-Seq reads were aligned to hg38 genome assembly using Tophat2 (v2.0.8) with default settings. Then, the gene expression levels were counted for obtaining raw counts with HTSeq (v0.6.1p1) against ensemble v86 annotation. The counts data were normalized using the trimmed mean of M values (TMM) method, implemented in the Bioconductor package “edgeR” to obtain the normalized RPKM (Reads Per Kilobase of transcript, per Million mapped reads) value. Genes were considered differentially expressed if fold change more than 2 or less than 0.5, *p* value <= 0.05 and at least one sample has RPKM >= 1. Hierarchical clustering of differentially expressed genes was performed using Cluster v3.0 with Pearson correlation distance and average linkage and visualized using Java Treeview. Enrichment analysis on Hallmark pathways was performed using Gene Set Enrichment Analysis (GSEA), implemented in the Bioconductor package “clusterProfiler.”

### 4.8. nCounter

Scrolls were obtained from HT29 tumor paraffin blocks. RNA extractions from molecular curls, nCounter and data analysis were performed at Molecular Pathology Core at City of Hope. Scrolls were first treated with Xylene followed by a wash with ethanol and RNA was extracted using miRNeasy FFPE kit (Qiagen cat# 217504), RNA concentration was assessed with the Nanodrop spectrophotometer ND-1000 and Qubit 3.0 Fluorometer (Thermo Fisher Scientific Inc, Waltham, MA, USA). RNA fragmentation and quality control were determined by 2100 Bioanalyzer (Agilent, Santa Clara, CA, USA). RNA expression was analyzed by NanoString nCounter platform (NanoString Technologies, WA, USA) using Human PanCancer Pathway panel. RNA was first hybridized with Codeset from gene panel at 65 °C for 16 h. The Post-hybridization probe-target mixture was then purified and quantified with nCounter Digital Analyzer. All raw data from expression analysis were first aligned with internal positive and negative controls, then normalized to the selected housekeeping genes included in the assay. Differential gene expression patterns as well as pathway scores with statistical analyses were performed with nSolver software (NanoString Technologies, Seattle, WA, USA). The data are presented on a log2 scale, in general, an increased score indicates increased overall expression.

### 4.9. Colony Assay

Five hundred cells of HT29 and HCT116 were seeded on 6 well-plates. The following day, cells were treated with 0.1% of EtOH, 100 nM and 500 nM VD, respectively and incubated for 10 days. Medium containing EtOH or VD was changed every 2–3 days. 10 days post-treatment, 500 μL 0.5% (*w/v*) crystal violet was added to each well and the plates were left at room temperature overnight. The next day, the plates were washed and scanned using Azure C600 scanner (Azure Biosystems, Dublin, CA, USA).

### 4.10. Immunofluorescence

To examine Ki 67 expression, HT29 and HCT116 cells treated with 0.1% EtOH, 100 nM and 500 nM of VD were compared to non-treated controls. Cells were incubated for 6 days with changing medium containing EtOH or VD every 2 days. Cells were fixed and permeabilized with cold methanol for 15 min at −20 °C and blocked for 30 min at room temperature in TNB blocking buffer (0.1M Tris-HCl, PH7.5, 0.15 NaCl and 0.5% Blocking Reagent-Perkin Elmer, cat FP1020). After blocking, cells were incubated with Rabbit anti-Ki67 antibody [SP6] (Cat# ab16667, Abcam, Cambridge, MA, USA) at 1:250 dilution in TNB blocking buffer and incubated for overnight at 4 °C. Cells were re-blocked in TNB blocking buffer, stained with secondary Goat Anti-Rabbit IgG H&L (Alexa Fluor^®^ 488) (Cat# ab150077, Abcam, Cambridge, MA, USA) at 1:500 dilution. Finally, 4′6-diamidino-2-phenylindole (DAPI) was added and images were obtained using EVOS FL Auto Imaging System (Thermo Fisher Scientific, Waltham, MA, USA).

### 4.11. Statistical Analysis

Statistical analysis was performed using GraphPad Prism (Version 7.01, La Jolla, CA, USA). Student’s t test or one-way ANOVA were used to evaluate for statistical significance. *p* < 0.05 was considered significant. Where present in figures, error bars indicate SD or SEM as defined in legends.

### 4.12. RT-PCR

Total RNA was extracted from tumors using the RNeasy Mini kit (Qiagen, Germantown, MD, USA). Next, cDNA was prepared from the total RNA using QuantiTect Reverse Transcription Kit (Qiagen, Germantown, MD, USA). GAPDH was used as an internal control. anti-PD-L1 mRNA were amplified using QuantiTect SYBR Green polymerase chain reaction (PCR) Kit (Qiagen, Germantown, MD, USA). Primer sequence were as following; Forward: GTAACGAGAGTACCCTGTCC, Reverse: ACACGAGTAAGAATACCGCC and Product size: 127bp.

### 4.13. Western Blotting

Protein was extracted from tumors. Next, 50 μg of each sample was separated on a Criterion Precast polyacrylamide gel (Cat# 3450028; BIO-RAD, Hercules, CA, USA) then transferred to a PVDF membrane. The membrane was stained with anti-DDDDK tag antibody (Cat# 1162; Abcam, Cambridge, MA, USA) to detect anti-PD-L1 FLAG tag and HRP-labeled goat anti-rabbit secondary antibody (Cat# ab205718; Abcam) diluted 1:1000 and 1:15,000, respectively in iBind Flex solutions (Cat# SLF2020; Invitrogen, Waltham, MA, USA) using iBind Flex Western Device (Thermo Fisher Scientific Inc, Waltham, MA, USA) for overnight. The membrane was then scanned using Azure C600 scanner (Azure Biosystems, Dublin, CA, USA).

## Figures and Tables

**Table 1:** Summary of Pathological Changes.

Pathological Changes	Control	Treated		
	PBS	OV	VD	VD + OV
**Necrosis**	+	++	++	+++
**Lymphocytes**	-	++	++	+
**Inflammatory cells**	+	+++	++	+
**cellular differentiation**	Moderate to poor	Moderate	Moderate	Moderate
**Vasculature**	+	+++	++	+
**Cancer-associated fibroblast**	+	++	+++	++

## Supplementary Materials

Supplemental materials can be found at https://www.mdpi.com/1422-0067/21/19/7326/s1.

## Abbreviations

CRC	Colorectal Cancer
MSI	Microsatellite instability
OV	Oncolytic Virus
PFU	Plaque-forming unit
TME	Tumor microenvironment
VD	Vitamin D
