# Supplementary figures and images for: Vitamin D as a Primer for Oncolytic Viral Therapy in Colon Cancer Models
Source: Int J Mol Sci. 2020 Oct 3;21(19):7326. doi: 10.3390/ijms21197326 (PMC7582493; doi:10.3390/ijms21197326)

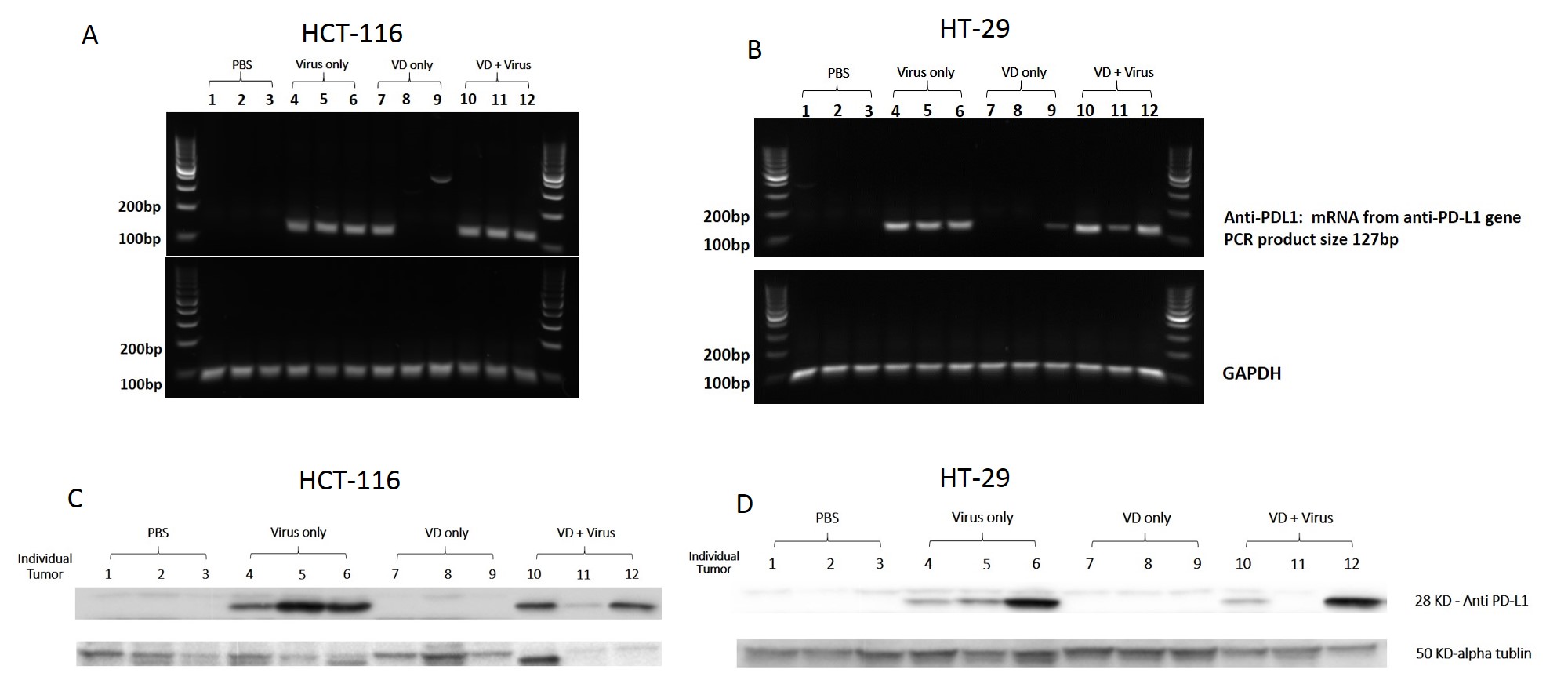

Supplement: Supplementary file 1 [file ijms-21-07326-s001.jpg]
